# A High-Throughput Screening System for *Populus* Wood-Associated Transcription Factors and Its Application to Lignin Regulation

**DOI:** 10.3389/fpls.2021.715809

**Published:** 2022-01-14

**Authors:** Yamei Zhuang, Sihui Chen, Wenjun Lian, Li Xu, Dian Wang, Congpeng Wang, Jie Meng, Xianfeng Tang, Hua Xu, Shumin Wang, Lin Du, Yang Zhang, Gongke Zhou, Guohua Chai

**Affiliations:** ^1^College of Resources and Environment, Qingdao Agricultural University, Qingdao, China; ^2^Qingdao Institute of BioEnergy and Bioprocess Technology, Chinese Academy of Sciences, Qingdao, China; ^3^College of Agronomy, Qingdao Agricultural University, Qingdao, China; ^4^Academy of Dongying Efficient Agricultural Technology and Industry on Saline and Alkaline Land in Collaboration With Qingdao Agricultural University, Dongying, China

**Keywords:** wood, transcription factors, Y1H, Y2H, lignin, *Populus*

## Abstract

Wood formation of trees is a complex and costly developmental process, whose regulatory network is involved in the protein-protein and protein-DNA interactions. To detect such interactions in wood development, we developed a high-throughput screening system with 517 Gal4-AD-wood-associated transcription factors (TFs) library from *Populus alba* × *P. glandulosa* cv “84K.” This system can be used for screening the upstream regulators and interacting proteins of targets by mating-based yeast-one hybrid (Y1H) and yeast-two-hybrid (Y2H) method, respectively. Multiple regulatory modules of lignin biosynthesis were identified based on this *Populus* system. Five TFs interacted with the 500-bp promoter fragment of *PHENYLALANINE AMMONIA-LYASE 2* (*PAL2*), the first rate-limiting enzyme gene in the lignin biosynthesis pathway, and 10 TFs interacted with *Pa*MYB4/LTF1, a key regulator of lignin biosynthesis. Some of these interactions were further validated by EMSA and BiFC assays. The TF-*PaPAL2* promoter interaction and TF-*Pa*MYB4 interaction revealed a complex mechanism governing the regulation of lignin synthesis in wood cells. Our high-throughput Y1H/Y2H screening system may be an efficient tool for studying regulatory network of wood formation in tree species.

## Introduction

The woody secondary cell walls of plants are the largest repository of renewable carbon biopolymers on the planet. They are widely used for timber, paper and pulp, and have potential as a source of bioenergy (Du and Groover, 2010). In tree species, wood (secondary xylem) is a complex biomass material constituted mainly of cellulose, hemicelluloses and lignin. Long cellulose microfibrils impart tensile strength, shorter hemicelluloses establish carbohydrate cross-linking, and lignin as a phenolic polymer fills in and cross-links the carbohydrate matrix (Albersheim et al., 2011). Understanding the molecular regulation of wood formation is required for the improvement of biomass and wood characteristics.

Wood formation is finely controlled by a hierarchical transcription factor network (HTFN), which is relatively conserved between Arabidopsis and trees (Wang and Dixon, 2012; Lin et al., 2013; Nakano et al., 2015; Chen et al., 2019). Functional characterization of a serial of Arabidopsis mutants shows a three-layered regulatory network for secondary cell wall formation (Zhong et al., 2010; Wang and Dixon, 2012; Nakano et al., 2015). In Arabidopsis stems, SND1 is the highest level regulator of HTFN, and it directly activates the second-layered master switches MYB46 and its paralog MYB83, inducing the expression of multiple cell wall biosynthetic genes. A wood-associated HTFN involving TF-DNA and TF-TF regulations has been recently constructed using quantitative transcriptomics, yeast one/two hybrid (Y1H/Y2H) and chromatin binding assays (Chen et al., 2019). Similar to Arabidopsis SND1-mediated HTFN, in *Populus* stems *Ptr*SND1-B1 induces *Ptr*MYB21, a homolog of Arabidopsis MYB46/83, to regulate the expression of a number of genes associated with cell wall component and wood biosynthesis. However, *Populus* undergoes multiple gene duplication events during evolutionary process, resulting in the ratio of 1.4∼1.6 *Populus* homologs to each Arabidopsis gene (Tuskan et al., 2006). Duplicated genes may have divergent fates such as subfunctionalization, neofunctionalization, or non-functionalization, causing more complex regulation of wood formation in *Populus* compared with Arabidopsis. For instance, the TF protein-complex regulators (dimers and trimers) are shown to cooperatively or combinatorially mediate the biosynthesis of specific types of lignin in *Populus* stems (Chen et al., 2019).

Lignin is the generic term for a large group of aromatic polymers, and is one of the most important limiting factors in the conversion of plant biomass to pulp or biofuels (Vanholme et al., 2010; Liu et al., 2014; Zhao, 2016). These lignin polymers are resulted from the oxidative combinatorial coupling of 4-hydroxyphenylpropanoids. In plants, the main biosynthetic route of lignin is generally conserved and involved in a battery of enzymes (Vanholme et al., 2010). PHENYLALANINE AMMONIA-LYASE (PAL) is the first rate-limiting enzyme in the lignin biosynthesis pathway, and catalyzes the deamination of L-phenylalanine to cinnamic acid (Bate et al., 1994). Five *Populus* PAL isoforms (*Ptr*PAL1 to *Ptr*PAL5) exhibit essentially identical catalytic activities in secondary differentiating xylem (SDX) based on the Michaelis-Menten kinetic parameters, indicating functional redundancy in the synthesis of monolignol (Wang et al., 2014). 4-COUMARATE:CoA LIGASE (4CL) is the last enzyme in the phenylpropionic acid pathway, activating the hydroxycinnamic acids to their corresponding esters with coenzyme A, a rate-limiting enzyme connecting the phenylpropionic acid pathway and the lignin biosynthesis pathway (Lu et al., 2004). In *Populus*, there are two 4CL isoforms (*Ptr*4CL3 and *Ptr*4CL5) with distinct reaction kinetic parameters (Chen et al., 2013). *Ptr*4CL3 has the highest conversion rate for 4-coumaric acid, while *Ptr*4CL5 most effectively metabolizes caffeic acid (Wang et al., 2014). Transcriptional regulation of *PtrPAL2* and *Ptr4CL3* expression were experimentally proved during lignin biosynthesis (Chen et al., 2019; Gui et al., 2019). In the *Populus* SDX protoplasts, the expression of *PtrPAL2* is directly activated by *Ptr*MYB21 in *Ptr*SND1-B1-mediated network (Chen et al., 2019). LTF1/*Pd*MYB4 was identified as an upstream regulator of *Pd4CL3* by screening a *Populus* developing xylem library using Y1H (Gui et al., 2019). In response to environmental stimuli, phosphorylation of LTF1 by MPK3/6 functions as a sensory switch regulating lignin biosynthesis. Currently, it remains unclear how *Ptr*PAL2 and LTF1/*Pd*MYB4, two lignin-associated proteins, are precisely regulated in woody cells.

To understand the regulatory mechanism of wood formation at the transcriptional level, we here generated a wood-associated Gal4-AD–TF library that contains 517 TFs from poplar, a fast-growing tree species. High-throughput Y1H and Y2H screens were applied to verify the efficiency of this library using two lignin-associated genes (*PaPAL2* and *LTF1/PaMYB4*) as the baits. The interactions between 5 TFs and the promoter fragment of *PaPAL2* and between 10 TFs and LTF1/*Pa*MYB4 offer a clue as to how lignin biosynthesis is precisely controlled. Our high-throughput Y1H/Y2H screening system is a powerful tool to help dissect transcriptional regulatory networks of wood formation in trees.

## Materials and Methods

### Construction of the Wood-Associated AD–TF Library

To generate the Gal4–AD-fused constructs, the coding regions of 517 TFs were amplified by PCR from the first-strand cDNA, that was prepared with the whole stem of 1.5-m-high *Populus alba* × *P. glandulosa* cv “84K” following the method described previously (Chai et al., 2014). After confirmation by sequencing, the PCR products were cloned into pGADT7 through the *Eco*RI site. The resulting constructs were transformed into the yeast strain Y187 using the PEG/LiAc method. The yeast cells containing the transformant was mixed with 30% sterilized glycerol in 2-ml 96-well plates and stored at −80°C for use.

### Mating-Based Yeast-One Hybrid and Yeast-Two-Hybrid Screening

The 500-bp promoter fragment of *PaPAL2* was amplified from genomic DNA of “84K” and the coding region of *LTF1/PaMYB4* was amplified from the xylem cDNA using gene-specific primers (Supplementary Table 1). Following the methods described by Ou et al. (2011), this *PaPAL2* promoter fragment was inserted into the vector pHIS2.1 for mating-based Y1H screening, and the *LTF1* coding region was inserted into pGBKT7 for mating-based Y2H screening. Yeast strain AH109 carrying the two baits was grown in selective medium and the Gal4–AD–TF strains were grown on SD-Leu medium in 96-well plates overnight. 30 μL/well of donor and host strains were transferred to a new 96-well plate with 100 μL YPAD medium in each well. Mating was conducted for 20–24 h by shaking at 30°C and 200 rpm. After 10-fold dilution with water, the mating products (5 μL well^–1^) were plated to different selective plates and incubated for 3 days at 30°C.

### Yeast-One Hybrid Assay

The coding regions of *PaMYB21*, *PaMYBH*, *PaWRKY20* and *PaDF1*, four regulatory candidates of *PaPAL2* identified by mating-based Y1H, were amplified from the xylem cDNA of “84K” (primers in Supplementary Table 1) and cloned into the pGADT7 vector. Each of these pGADT7-TFs (pGADT7 as control) and pHIS-*proPaPAL2* were co-transformed into yeast stain Y187. The transformed cells were observed on the SD/-Leu-His plates and triple dropout plates (SD/-Trp-His-Leu) supplemented with 10 mM 3-amino-1,2,4-triazole (3-AT) for 3 days at 28°C, following the Y1H procedure described previously (Wang et al., 2020).

### EMSA

EMSA was performed following the method described previously (Chai et al., 2014). The coding regions of *PaMYBH*, *PaWRKY20*, and *PaDF1* were fused in frame with MBP in pMAL-p4X and expressed in *Escherichia coli*. Recombinant protein was purified using amylose resin (New England Biolabs). The *PaPAL2* promoter fragments covering the corresponding binding sites of *PaMYBH*, *PaWRKY20*, and *PaDF1* were labeled with biotin at the 5’ end and used as the probes (Supplementary Figure 1, BGI). The same unlabeled oligos were annealed for competition. Binding reactions were performed with a LightShift® Chemiluminescent EMSA Kit (Thermo Fisher Scientific) according to the manufacturer’s instructions. The *Pa* MYBH-, *Pa*WRKY20- or *Pa*DF1-bound DNA fragments were separated from the unbound fragments by polyacrylamide gel electrophoresis. The DNA on a nitrocellulose membrane was detected by chemiluminescence. Three independent experiments were performed for each probe.

### Yeast-Two-Hybrid Assay

The coding regions of *PaMYB21*, *PaGRAS2*, *PaWRKY20*, *PaANAC83*, and *PaDF1* identified by mating Y2H were amplified from the xylem cDNA of “84K” and cloned into the pGBKT7 vector (BD). Primers were shown in Supplementary Table 1. The pGADT7-LTF1/*Pa*MYB4 and pGBKT7-TF were co-transformed into yeast strain AH109. The transformed cells were cultured on SD/-Leu/-Trp plates for 2 days, and then dropped on the SD/-Leu/-Trp/-Ade/-His medium with or without 5 mM 3-AT for 3 days at 30°C following the Y2H procedure described previously (Tang et al., 2020). The empty vector pGADT7 was used as blank control.

### BiFC

Bimolecular fluorescence complementation (BiFC) assays were conducted according to the method described previously (Tang et al., 2020). The coding region of *LTF1/PaMYB4* was cloned into the pSAT1-nVenus (pE3228) vector, and that of *PaMYB21*, *PaWRKY20* or *PaANAC83* was cloned into pSAT1-cCFP (pE3242) (Walter et al., 2004). Arabidopsis leaf protoplasts were isolated and transfected following the method established by Sheen (2001). Confocal microscopy was performed with an Olympus FluoView FV1000 confocal microscope using an excitation wavelength of 488 nm and detection at 499–535 nm. The experiments were repeated three times.

## Results

### Construction of a Wood-Associated Transcription Factor Library

**FIGURE 1:**
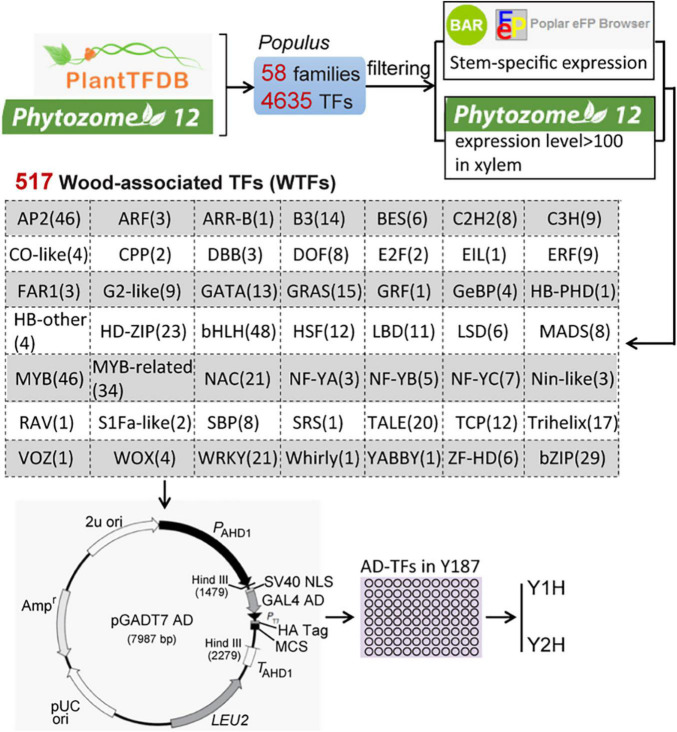
The scheme of the construction of a wood-associated transcription factors (TFs) library. A total of 517 Wood-associated TFs (WTFs) were identified from 4287 *Populus* TFs of 58 gene families in two database (PlantTDB and Phytozome 12) using the stringent filtering criteria. All pGADT7-TFs were independently transformed into the yeast strain Y187 for throughout mating-based Y1H or Y2H screening.

To obtain wood-associated transcription factors (WTFs), we extracted 4287 *Populus* TFs covering 58 gene families from both PlantTFDB^1^ and Phytozome 12^2^ (Figure 1). A total of 517 genes in 49 families were defined as WTFs based on the stringent filtering criteria as follows: genes show high (> 100) expression levels in xylem in Phytozome 12 and specific expression in wood tissues identified by the online software BAR^3^. Full-length ORFs of the 517 genes were amplified from the first-strand cDNA in the whole stems of *Populus alba* × *P. glandulosa* cv “84K.” After validation by sequencing, All ORFs were independently inserted into the pGADT7 vector through the EcoRI site at multiple clone sites (MCS) and then transformed into the yeast strain Y187.

### Identification of Transcription Factor That Binds to the *PaPAL2* Promoter

**FIGURE 2:**
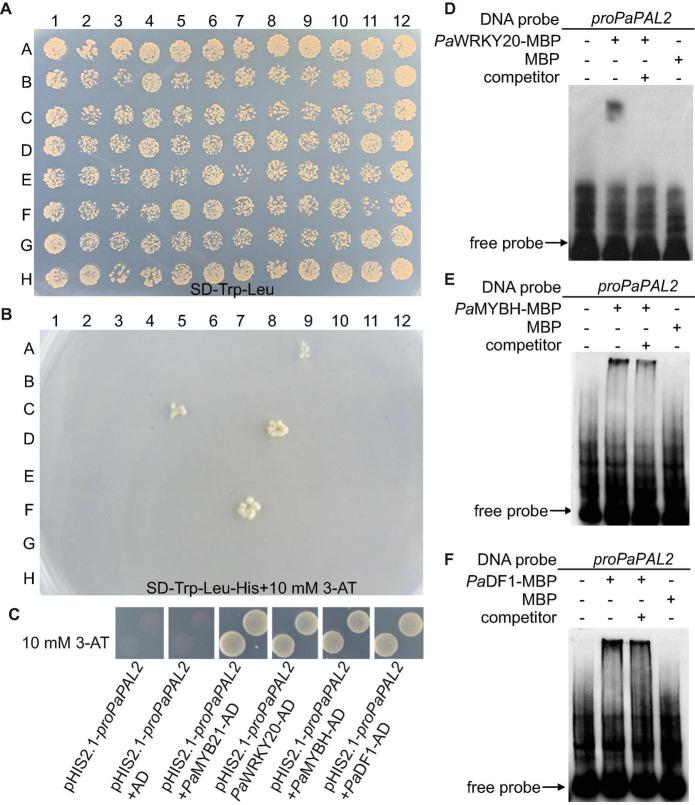
Identification of TFs that bind to the *PaPAL2* promoter. **(A,B)** Y1H screening of the TFs library using 500-bp *PaPAL2* promoter fragment as a bait. Yeast growth on the non-selective medium SD-Trp-Leu showed the mating efficiency. The candidate TFs can be detected on medium with 10 mM 3-AT. **(C)** Y1H assays verifying that *Pa*MYB21, *Pa*WRKY20, *Pa*MYBH, or *Pa*DF1 was able to bind to the *PaPAL2* promoter fragment. Plasmids transformed into Y187 were screened on the SD/-Trp-Leu-His medium with 10 mM 3-AT. **(D–F)** EMSA results showing the specific bindings of *Pa*WRKY20, *Pa*MYBH, or *Pa*DF1 protein to the *PaPAL2* promoter *in vitro*. For competition assays, unlabeled probes (competitor) in 20-fold (+) molar excess relative to the labeled probe was include in the reaction.

*PaPAL2*, the ortholog of Arabidopsis *PAL1* in *Populus*, is a rate-limiting enzyme that controls the deamination of phenylpropanoid to cinnamic acid during monolignol biosynthesis in woody cells (Wang et al., 2014). Further, *PaPAL2* is a direct target of *Pa*MYB21 that is the second-layered master regulator of wood formation (Chen et al., 2019). Thus, *PaPAL2* was selected to test the efficiency of this yeast library and to clarify the lignin biosynthesis pathway. Following the method described previously (Ou et al., 2011), we screened this library by mating the bait, a 500 bp promoter fragment upstream of ATG of *PaPAL2*, in Y1H assays. Yeast cells co-transformed with pHIS-*proPaPAL2* and each of five pGADT7-TFs that were identified were able to grow normally on medium SD-Trp-Leu-His with 10 mM 3-AT (Figures 2A,B). These five WTFs belong to the MYB (*Pa*MYB21), HD-ZIP (*Pa*HB5), MYB-related (*Pa*MYBH), Trihelix (*Pa*DF1), and WRKY (*Pa*WRKY20) family (Table 1). We further examined the cell-type expression patterns of *PaPAL2* and its five regulatory candidates in stems, based on high-spatial-resolution transcriptome data that were sampled across secondary stem tissues in *Populus* (Sundell et al., 2017). As revealed in Supplementary Figure 2A, *PaPAL2*, *PaMYB21*, *PaMYBH*, *PaHB5*, *PaDF1*, or *PaWRKY20* were expressed in wood-forming cells, including the cambial zone and secondary phloem/xylem cells.

**TABLE 1:** Transcription factors that bind to the *PaPAL2* promoter identified in the *Populus* wood-associated library using Y1H.

No.	Loci	Name	Arabidopsis homolog	Gene family
1	Potri.009G053900.1	*Pa*MYB21	MYB83	MYB family
2	Potri.002G068600.1	*Pa*DF1	DF1	Trihelix family
3	Potri.005G071900.1	*Pa*HB5	HB5	HD-ZIP family
4	Potri.001G189800.1	*Pa*MYBH	MYBH	MYB-related family
5	Potri.001G361600.1	*Pa*WRKY20	WRKY20	WRKY family

*Pa*MYB21, *Pa*DF1, *Pa*MYBH, and *Pa*WRKY20, like *PaPAL2*, exhibited high expression in lignified xylem, and they were selected for validation by Y1H method. Yeast cells co-transformed with pHIS-*proPaPAL2* and pGADT7-TF grew normally on the SD-Trp-Leu-His medium supplemented with 10 mM 3-AT (Figure 2C), indicating the binding of *Pa*MYB21, *Pa*DF1, *Pa*MYBH or *Pa*WRKY20 to the *PaPAL2* promoter in yeast. Analysis of the 500-bp *PaPAL2* promoter fragment showed the DNA-binding motifs of *Pa*DF1 (GT, Kaplan-Levy et al., 2012), *Pa*MYBH (MRE, Du et al., 2013) and *Pa*WRKY20 (W-box, Ulker and Somssich, 2004; Supplementary Figure 2). Not surprisingly, EMSA results revealed that *Pa*DF1, *Pa*MYBH, and *Pa*WRKY20 proteins were able to bind to these cis-elements of the *PaPAL2* promoter specifically *in vitro* (Figures 2D–F).

### Identification of Transcription Factor That Interacts With *Pa*MYB4/LTF1

**FIGURE 3:**
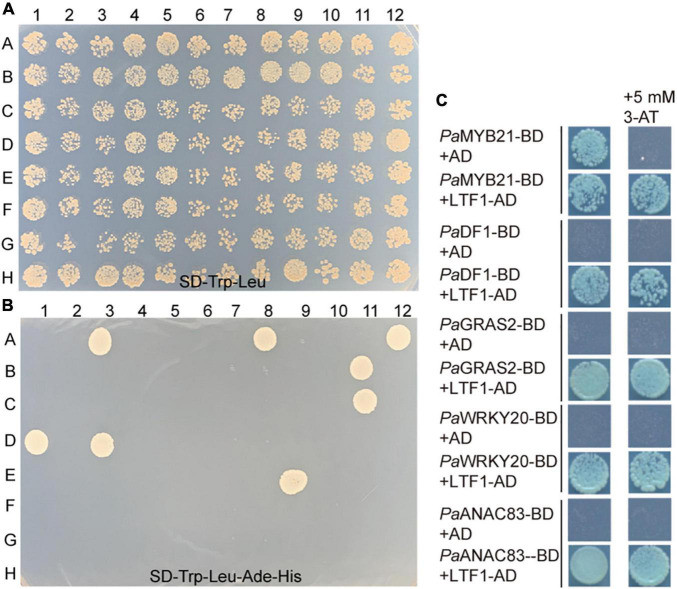
Identification of TFs that interact with *Pa*MYB4/LTF1. **(A,B)** Y2H screening of this TFs library by mating the bait *Pa*MYB4/LTF1. Strong interaction can be detected on the stringent medium SD-Leu-Trp-His-Ade. **(C)** Y2H assays in yeast confirming the interaction between *Pa*MYB4/LTF1 and *Pa*MYB21, *Pa*DF1, *Pa*GRAS2, *Pa*WRKY20, or *Pa*ANAC83 *in vivo*. Plasmids transformed into AH109 were screened on the SD/-Trp-Leu-His-Ade medium with or without 5 mM 3-AT.

LTF1/*Pa*MYB4 is a key negative regulator of lignin biosynthesis in wood formation (Gui et al., 2019; Holwerda et al., 2019). To elucidate the regulatory mechanism of lignin biosynthesis, we screened the LTF1/*Pa*MYB4-interacting proteins in our TF library by mating the bait LTF1/*Pa*MYB4. Ten TF clones were obtained (Figures 3A,B). Three of them were in the Trihelix family, three were in the GRAS family, and one in the MYB, NAC, WRKY, or SBP family (Table 2). Interestingly, these TFs included *Pa*MYB21, *Pa*DF1, and *Pa*WRKY20, that were shown to directly regulate *PaPAL2* expression (Figure 2). Analysis of high-spatial-resolution wood transcriptome profiles revealed the high expression of LTF1/*Pa*MYB4 and its 10 interacting proteins in wood-forming tissues such as secondary phloem and lignified xylem (Supplementary Figure 1B).

**TABLE 2:** Transcription factors that interact with *Pa*MYB4/LTF1 identified in the *Populus* wood-associated library using Y2H.

No.	Loci	Name	Arabidopsis homolog	Family
1	Potri.009G053900.1	*Pa*MYB21	MYB83	MYB family
2	Potri.002G068600.1	*Pa*DF1	DF1	Trihelix family
3	Potri.001G113600.1	Potri.001G113600	AT3G58630	Trihelix family
4	Potri.003G195300.1	Potri.003G195300	AT3G54390	Trihelix family
5	Potri.007G132000.1	*Pa*SHR	SHR	GRAS family
6	Potri.009G033300.1	*Pa*GRAS2	GRAS2	GRAS family
7	Potri.005G123800.1	Potri.005G123800	AT5G66770	GRAS family
8	Potri.001G361600.1	*Pa*WRKY20	WRKY20	WRKY family
9	Potri.001G061200.1	*Pa*ANAC83	ANAC083	NAC family
10	Potri.002G002400.1	Potri.002G002400	AT1G76580	SBP family

**FIGURE 4:**
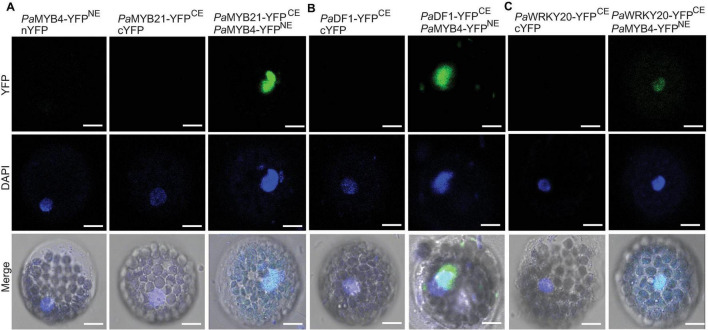
BiFC validation of the *in vivo* interaction between *Pa*MYB21, *Pa*DF1, or *Pa*WRKY20 and *Pa*MYB4/LTF1. **(A–C)** *Pa* MYB21-, *Pa* DF1-, or *Pa*WRKY20-YFP^CE^ and *Pa*MYB4-YFP^NE^ interact to form a functional YFP in the nucleus of *Arabidopsis* leaf protoplasts. The DNA fluorochrome 4′, 6-diamidino-2-phenylindole (DAPI) was used to stain the cell nucleus (blue). Bar = 10 μm.

Five TFs (*Pa*MYB21, *Pa*DF1, *Pa*GRAS2, *Pa*WRKY20, and *Pa*ANAC83) were selected to verify their interactions with *Pa*MYB4 using Y2H (Figure 3C). Yeast cells transformed with TF-BD and *Pa*MYB4-AD exhibited blue on SD/-Leu-Trp-His-Ade plate, confirming the interaction between the five TFs and LTF1/*Pa*MYB4. Of the five interacting TFs, only *Pa*MYB21 protein had self-activation in yeast, that can be effectively inhibited by addition of 5 mM 3-AT. BiFC assays were conducted in Arabidopsis leaf protoplasts to verify the *in vivo* interaction between *Pa*MYB4/TFL1 and *Pa*MYB21, *Pa*DF1, or *Pa*WRKY20 (Figure 4). Co-expression of *Pa*MYB4/LTF1 fused to the amino-terminal half of YFP (YFP^NE^) and *Pa*MYB21, *Pa*DF1, or *Pa*WRKY20 fused to the carboxy-terminal half (YFP^CE^) of yellow florescent protein led to visible fluorescence in the nucleus of co-transformed protoplasts. However, no YFP fluorescence was detected when *Pa*MYB4-YFP^NE^ was co-expressed with the carboxy-terminal half of YFP (cYFP) or *Pa*MYB21-, *Pa*DF1- or *Pa*WRKY20-YFP^CE^ was co-expressed with the amino-terminal half of YFP (nYFP).

## Discussion

Recent studies in the stem of *Populus* show that wood formation involves regulatory homeostasis determined by combinations of TF-DNA and TF-protein regulations (Petzold et al., 2017; Chen et al., 2019). Using Y1H and Y2H assays, 40 protein-DNA interactions involving 20 different TFs and 165 protein-protein interactions involving 162 different proteins are shown to be relevant to wood formation (Petzold et al., 2017). These interactions are incorporated into a network that includes 14 connected subnetworks, with the largest having 132 members. Integration of quantitative transcriptomics and chromatin binding data constructs a TRN, in which *Ptr*SND1-B1 directs 57 TF-DNA interactions through 17 TFs transregulating 27 cell wall genes (Chen et al., 2019). Of the multiple methods for determining TF-DNA and TF-protein interactions, Y1H and Y2H screenings are the most widely used in trees. However, a major limitation for the two methods is low expression levels of TFs in wood-forming tissues. To overcome this disadvantage, we set up a high throughout mating-based screening system that includes a Gal4-AD-TF library of 517 wood-associated TFs (WTFs). The 517 WTFs were filtered by the stringent criteria and accounted for 11% of all *Populus* TFs (4287). By mating yeast strains (bait) with a series of strains (prey) expressing WTF-AD protein, we were able to identify protein-DNA or protein-protein interactions and identify connected networks during wood formation. Importantly, our system is still effective for Y1H screening using a single fragment of up to 500 bp as a bait, which has an advantage of minimizing the effort to find out the exact cis-element and increasing the screening specificity. Thus, our library is a resource for Y1H and Y2H assay and functional analyses of genes associated with poplar wood formation.

Lignin biosynthesis starts with the deamination of phenylalanine, followed by a series of hydroxylation, methylation and reduction resulting in the production of p-hydroxyphenyl (H), guaiacyl (G), and syringyl (S), three basic units of the lignin complex in trees (Zhao, 2016). In the *Populus* lignin biosynthesis pathway, *Ptr*MYB90, *Ptr*MYB161, *Ptr*NAC123, or *Ptr*WBLH1 targets G-specific *CCoAOMT* gene family members, and *Ptr*MYB90, *Ptr*MYB161, or *Ptr*WBLH1 targets *CAld5H* that is needed for the biosynthesis of the S subunits (Chen et al., 2019). In this study, we focus on *Pa*PAL2 that is rate-limiting enzyme in the lignin synthesis pathway, and LTF1/*Pa*MYB4 that targets *Pa*4CL3, a key enzyme of general phenylpropanoid metabolism that provides the precursors for both lignin biosynthesis (Lu et al., 2004; Chen et al., 2013, 2019; Wang et al., 2014; Gui et al., 2019).

With the help of a high-throughput Y1H/Y2H screening system, we identified five TFs that bound to the *PaPAL2* promoter fragment and 10 TFs interacting with *Pa*MYB4 in *Populus*, indicating a high efficiency of our library. Selected interactions were further verified by EMSA and BiFC independently. Of these TFs, *Pa*MYB21, *Pa*WRKY20, *Pa*DF1 were found to interact with the *PaPAL2* promoter or *Pa*MYB4 protein. Consistently, they exhibited overlapping expression with *PaPAL2* or *PaMYB4* in wood-forming tissues predicted by high-spatial-resolution wood transcriptome data (Sundell et al., 2017). Of these interactors, *Ptr*MYB21 is proven to directly activate *PtrPAL2* expression, promoting lignin biosynthesis during wood formation (Chen et al., 2019). Other TFs are not functionally characterized in trees, but the homologs of *Pa*DF1 and *Pa*WRKY20 in Arabidopsis were identified as the components of the gene regulatory network for secondary cell wall synthesis in stems (Taylor-Teeples et al., 2015). These preliminary results revealed a coordinated role of the TF-*proPaPAL2* and TF-*Pa*MYB4 interactions in lignin biosynthesis in *Populus* stems. The TF-DNA interactions for *Populus PAL4*, a paralog of *PaPAL2*, were also identified in xylem cells by Y1H assays (Petzold et al., 2017). Six TFs, including *Ptr*MYB2 that is a homolog of *Ptr*MYB21, are shown to bind to the *PtrPAL4* promoter. This suggests different TF-DNA interactions between homologs in woody cells. Our powerful system will facilitate to generate comprehensive interaction networks of TFs to understand the regulatory mechanism of wood formation in tree species.

## Data Availability Statement

The original contributions presented in the study are included in the article/Supplementary Material, further inquiries can be directed to the corresponding author/s.

## Author Contributions

GC designed the experiments, performed data processing, and drafted the manuscript. YZhu, SC, WL, LX, DW, CW, JM, XT, HX, SW, LD, and YZha prepared the materials and performed the experiments. GZ conceived the study and revised the manuscript. All authors read and approved the final version of the manuscript.

## Conflict of Interest

The authors declare that the research was conducted in the absence of any commercial or financial relationships that could be construed as a potential conflict of interest.

## Publisher’s Note

All claims expressed in this article are solely those of the authors and do not necessarily represent those of their affiliated organizations, or those of the publisher, the editors and the reviewers. Any product that may be evaluated in this article, or claim that may be made by its manufacturer, is not guaranteed or endorsed by the publisher.

## Footnotes

1
http://planttfdb.gao-lab.org/

2
https://phytozome.jgi.doe.gov/pz/portal.html

3
http://bar.utoronto.ca/efppop/cgi-bin/efpWeb.cgi
