# Supplementary material for: A High-Throughput Screening System for Populus Wood-Associated Transcription Factors and Its Application to Lignin Regulation
Source: Front Plant Sci. 2022 Jan 14;12:715809. doi: 10.3389/fpls.2021.715809 (PMC8795814; doi:10.3389/fpls.2021.715809)
Supplement: Supplementary file 1 [file Data_Sheet_1.docx]

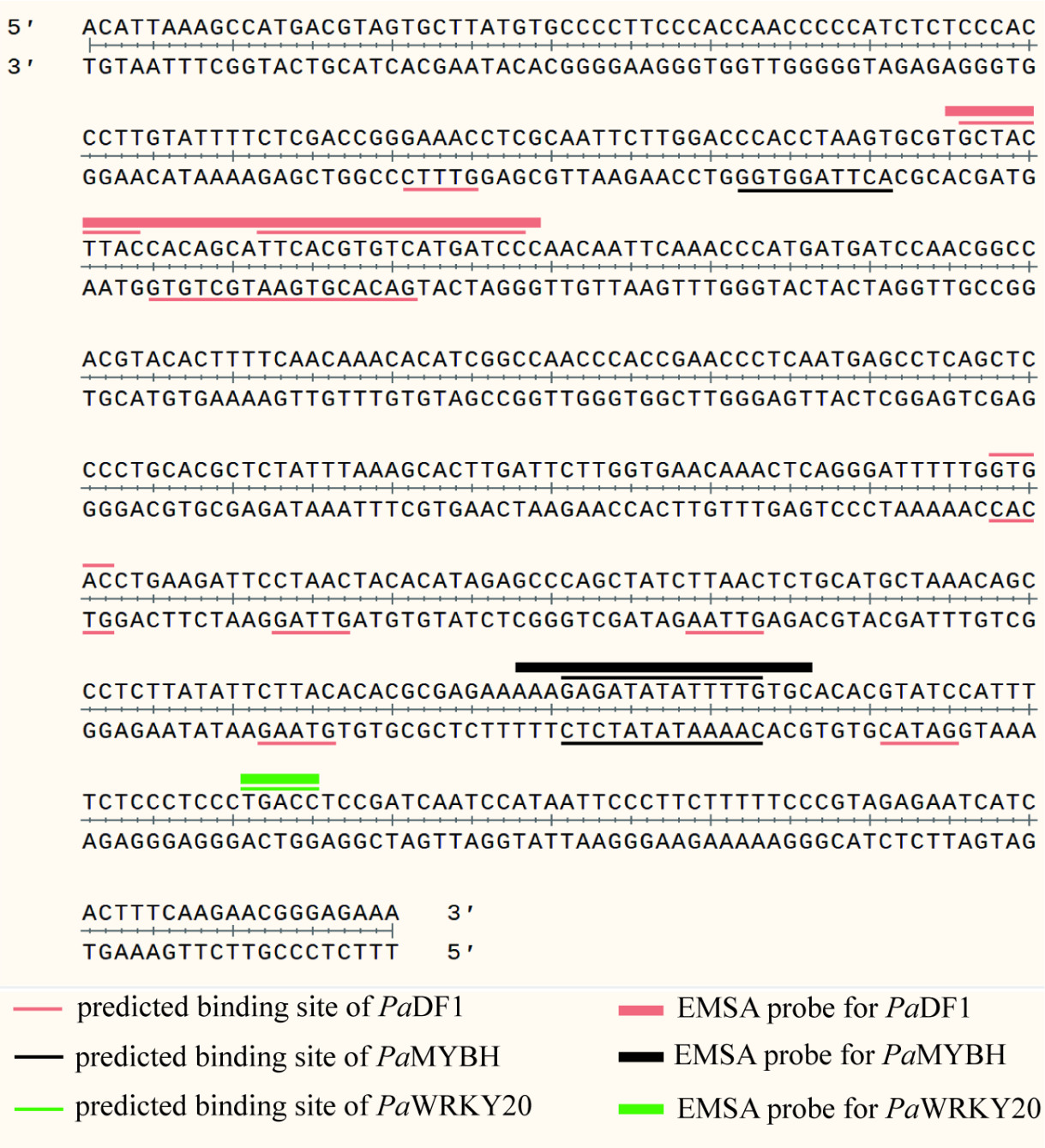


**Figure S1. Binding sites of *Pa*MYBH, *Pa*WRKY20 or *Pa*DF1 protein in a 500-bp fragment of *PaPAL2* promoter**

**
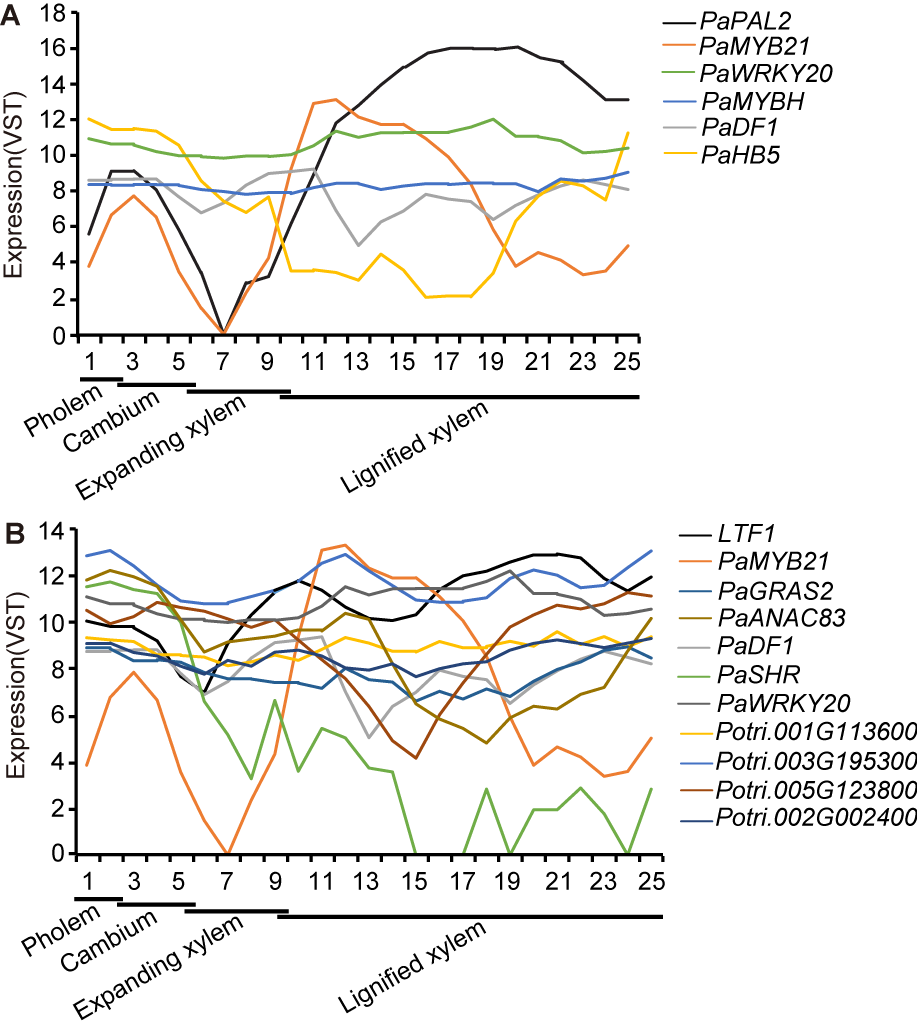
**

**Figure S2. Expression patterns of *PaPAL2* and its associated genes in stems based on the RNA-seq data of *Populus.***

The curves were generated based on the values extracted from the RNA-seq datasets of *Populus* stems (Sundell et al*.*, 2017). (**A**) Expression patterns of *PaPAL2* and its five upstream regulators across secondary stem tissues. (**B**) Expression patterns of *Pa*MYB4**/**LTF1 and its ten interacting proteins across secondary stem tissues.

**Table S1. Primers used in this study**

| Gene | Locus | | Primers | Sequence (5’-3’) | Goal |
| --- | --- | --- | --- | --- | --- |
| *PaPAL2* | Potri.008G038200 | | PaPAL2-pHIS2.1-F | ctatagggcgaattcCGACACGCGATTGCAGTGTAACAC | For amplifying *PaPAL2* promoter to generate the Gal4-AD vector for Y1H analysis |
|  |  |  | PaPAL2-pHIS2.1-R | atccacgcgtgagctcTTTCTCCCGTTCTTGAAAGTGA |  |
| *PaLTF1* | Potri.004G174400 | | LTF1-BD-F | AATTCCCGGGGATCCCCATGGGAAGGTCTCCTTGC | For amplifying *PaLTF1* to generate the Gal4-BD vector for Y2H analysis |
|  |  |  | LTF1-BD-R | CAGGTCGACGGATCCTCATTTCATCTCCAAACCTCTATAATCC |  |
|  |  |  | LTF1-3228-F | GCGGGCCCGGGATCCATGGGAAGGTCTCCTTGC | For amplifying *PaLTF1* to generate  the C end of YFP vector for BIFC analysis |
|  |  |  | LTF1-3228-R | TAGATCAGGTGGATCCTCATTTCATCTCCAAACCTCTATAATCC |  |
|  |  |  | LTF1-AD-F | GCCATGGAGGCCAGTGAATTCATGGGAAGGTCTCCTTGC | For amplifying *PaLTF1* to generate the Gal4-AD vector for Y1H/Y2H analysis |
|  |  |  | LTF1-AD-R | ATGCCCACCCGGGTGGAATTCTCATTTCATCTCCAAACCTCTATAATCC |  |
| *PaMYB21* | Potri.009G053900 | | PaMYB21-AD-F | GCCATGGAGGCCAGTGAATTCATGAGGAAGCCAGAGGC | For amplifying *PaMYB21* to generate the Gal4-AD vector for Y1H/Y2H analysis |
|  |  |  | PaMYB21-AD-R | ATGCCCACCCGGGTGGAATTCTCATTGGAAATCAAGGAATGGAAAG |  |
|  |  |  | PaMYB21-BD-F | AATTCCCGGGGATCCCCATGAGGAAGCCAGAGGC | For amplifying *PaMYB21* to generate the Gal4-BD vector for Y2H analysis |
|  |  |  | PaMYB21-BD-R | CAGGTCGACGGATCCTCATTGGAAATCAAGGAATGGAAAG |  |
|  |  |  | PaMYB21-3242-F | CGCGGGCCCGGGATCCATGAGGAAGCCAGAGGC | For amplifying *PaMYB21* to generate the N end of YFP vector for BIFC analysis |
|  |  |  | PaMYB21-3242-R | CTCTAGATCAGGTGGATCCTCATTGGAAATCAAGGAATGGAAAG |  |
| *PaMYBH* | Potri.001G189800 | | PaMYBH-AD-F | GCCATGGAGGCCAGTGAATTCATGACTCGGCGGTGTT | For amplifying *PaMYBH* to generate the Gal4-AD vector for  Y1H/Y2H analysis |
|  |  |  | PaMYBH-AD-R | ATGCCCACCCGGGTGGAATTCTCAAACTGCTTGGATGGGAC |  |
|  |  |  | PaMYBH-MBP-F | GAATTCGGATCCATGACTCGGCGGTGTT | For amplifying *PaMYBH* to generate MBP confusion protein |
|  |  |  | PaMYBH-MBP-R | TCTAGAGGATCCTCAAACTGCTTGGATGGGAC |  |
| *PaWRKY20* | | Potri.001G361600 | PaWRKY20-AD-F | GCCATGGAGGCCAGTGAATTCATGGACAGCAATACCTCTCG | For amplifying *PaWRKY20* to generate the Gal4-AD vector for Y1H/Y2H analysis |
|  |  |  | PaWRKY20-AD-R | ATGCCCACCCGGGTGGAATTCTTATGGACCCGTTAATAATCTTCCCA |  |
|  |  |  | PaWRKY20-BD-F | AATTCCCGGGGATCCCCATGGACAGCAATACCTCTCG | For amplifying *PaWRKY20* to generate the Gal4-BD vector for Y2H analysis |
|  |  |  | PaWRKY20-BD-R | CAGGTCGACGGATCCTTATGGACCCGTTAATAATCTTCCCA |  |
|  |  |  | PaWRKY20-3242-F | CGCGGGCCCGGGATCCATGGACAGCAATACCTCTCG | For amplifying *PaWRKY20* to generate the N end of YFP vector for BIFC analysis |
|  |  |  | PaWRKY20-3242-R | CTCTAGATCAGGTGGATCCTTATGGACCCGTTAATAATCTTCCCA |  |
|  |  |  | PaWRKY20-MBP-F | GAATTCGGATCCATGGACAGCAATACCTCTCG | For amplifying *PaWRKY20* to generate MBP confusion protein |
|  |  |  | PaWRKY20-MBP-R | TCTAGAGGATCCTTATGGACCCGTTAATAATCTTCCCA |  |
| *PaDF1* | | Potri.002G068600 | PaDF1-AD-F | GCCATGGAGGCCAGTGAATTCATGCTAGGGGACTCAAGTAGT | For amplifying *PaFF1* to generate the Gal4-AD vector for Y1H/Y2H analysis |
|  |  |  | PaDF1-AD-R | ATGCCCACCCGGGTGGAATTCTCACCCGGCTGTGTTCA |  |
|  |  |  | PaDF1-BD-F | AATTCCCGGGGATCCCCATGCTAGGGGACTCAAGTAGT | For amplifying *PaDF1* to generate the Gal4-BD vector for Y2H analysis |
|  |  |  | PaDF1-BD-R | CAGGTCGACGGATCCTCACCCGGCTGTGTTCA |  |
|  |  |  | PaDF1-MBP-F | GAATTCGGATCCATGCTAGGGGACTCAAGTAGT | For amplifying *PaDF1* to generate MBP confusion protein |
|  |  |  | PaDF1-MBP-R | TCTAGAGGATCCTCACCCGGCTGTGTTCA |  |
| *PaGRAS2* | Potri.009G033300 | | PaPaGRAS2-BD-F | AATTCCCGGGGATCCCCATGGAGAAGCTTAGTTTTGTTAAGAAT | For amplifying *PaGRAS2* to generate the Gal4-BD vector for Y2H analysis |
|  |  |  | PaPaGRAS2-BD-R | CAGGTCGACGGATCCCTATGGTTTTCTTCTAACGTAAGGAAAA |  |
| *PaANAC83* | Potri.001G061200 | | PaANAC83-BD-F | AATTCCCGGGGATCCCCATGGAGAAGCTTAGTTTTGTTAAGAAT | For amplifying *PaANAC83* to generate the Gal4-BD vector for Y2H analysis |
|  |  |  | PaANAC83-BD-R | CAGGTCGACGGATCCCTATGGTTTTCTTCTAACGTAAGGAAAA |  |
|  |  |  | PaANAC83-3242-F | CGCGGGCCCGGGATCCATGGAGAAGCTTAGTTTTGTTAAGAAT | For amplifying *PaANAC83* to generate the N end of YFP vector for BIFC analysis |
|  |  |  | PaANAC83-3242-R | CTCTAGATCAGGTGGATCCCTATGGTTTTCTTCTAACGTAAGGAAAA |  |
